# Buffalo City Medical Association

**Published:** 1851-07

**Authors:** 


*Buffalo City Medical Association.*—We have inadvertently omitted heretofore to insert the officers of this Association for the present year. They are as follows:

| | |
|---|---|
| President, . . . . | Charles W. Harvey, M. D. |
| Vice President, . . . | S. Hubbard, M. D. |
| Recording Secretary, . . | James S. Hawley, M. D. |
| Primary Board, . . . | E. Wallis, M. D., Wm. Treat, M. D., C. C. Wychoff, M. D. |

The meetings of the Association are held semi-monthly, instead of once in each month as has formerly been the case.

